# High-performance broadband SiC nanoparticles/porous silicon photodetector prepared by laser ablation in liquid

**DOI:** 10.1039/d6ra01293k

**Published:** 2026-07-07

**Authors:** Shahad J. Neamah, Raid A. Ismail, Mehdi Q. Zayer, Khawla S. Khashan

**Affiliations:** a College of Applied Science, University of Technology Iraq raidismail@yahoo.com

## Abstract

Improving the figures of merit of heterojunction photodetectors has attracted significant research attention due to the growing demand for high-performance optoelectronic devices in advanced industrial and technological applications. The integration of nanomaterials with a porous silicon structure substrate offers an effective approach to enhance the light absorption, carrier separation efficiency, and interfacial charge transport in silicon-based photodetectors. Here, we report the fabrication of a SiC nanoparticles/porous silicon heterojunction photodetector. The SiC nanoparticles (NPs) are synthesized by laser ablation of a silicon target in ethanol, and porous silicon (PSi) is fabricated by electrochemical anodization. The X-ray diffraction studies reveal that the synthesized SiC nanoparticles are crystalline in nature with a hexagonal structure (α-SiC phase). The energy gap of the SiC colloidal nanoparticles was found to be 3.75 eV. Scanning electron microscopy (SEM) investigations showed the formation of spherical SiC nanoparticles with an average particle size of 65.5 ± 18 nm. The electrical properties and photoresponse of the SiC/(n,p) PSi photodetectors were investigated. The responsivity and detectivity of the SiC NPs/p-PSi photodetector were 1.44 A W^−1^ and 3.07 × 10^11^ Jones at 450 nm, respectively, while SiC NPs/n-PSi exhibited a responsivity of 0.9 A W^−1^ and a detectivity of 1.83 × 10^11^ Jones at 450 nm. The photocurrent-time-dependent results revealed that the rise time/fall time ratios (*τ*_r_/*τ*_f_) of SiC NPs/p-PSi and SiC NPs/n-PSi photodetectors were 248 ms/306 ms and 168.8 ms/190.9 ms, respectively, and the corresponding ON/OFF current ratio was 137 and 30.

## Abstract 2

Improving the figures of merit of heterojunction photodetectors has attracted significant research attention due to the growing demand for high-performance optoelectronic devices in advanced industrial and technological applications.

## Graphical abstract

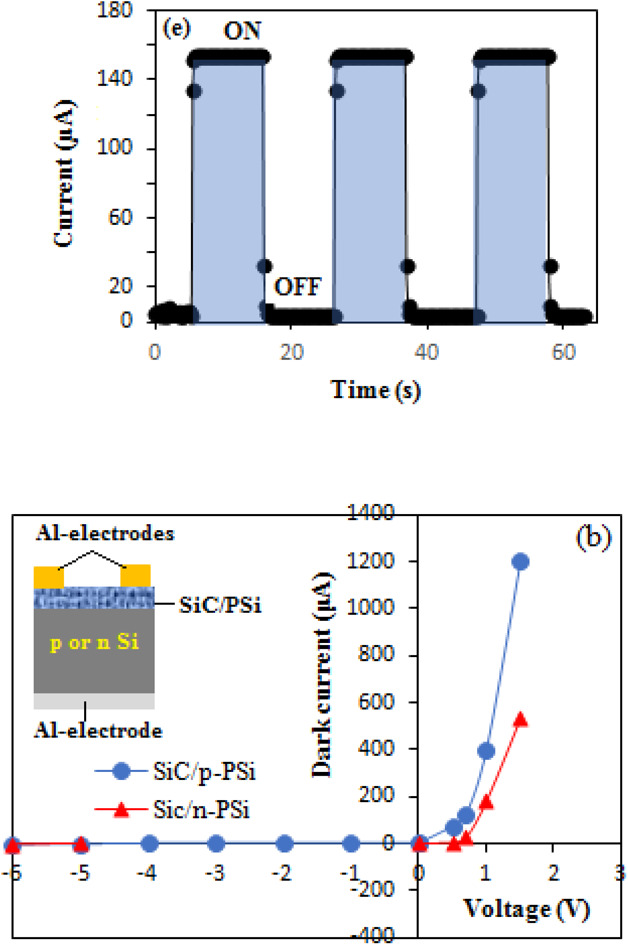

## 1. Introduction

There remains great interest in improving the optical, electrical, and photoresponse properties of photodetectors for use in many advanced industrial and technological applications. Carbide-on-carbide (SiC) nanoparticles are considered one of the functional materials because they possess superior properties, such as a wide optical energy gap and good thermal conductivity ^1,2^, high electron mobility, the ability to withstand harsh environments, high corrosion resistance, chemical stability, and high hardness.^3^ The SiC nanomaterials were found to exhibit high luminous efficiency that comes from the quantum confinement effect, and this approach has found efficient applications in biological imaging and solid-state lighting.^4–6^ As reported, there are many types of SiC nanoparticles, such as 3C–SiC, 4H–SiC, and 6H–SiC, where the energy gap for these types is approximately 2.3, 3.2, and 3.03 eV, respectively.^7^ Numerous methods have been adopted to prepare the SiC nanoparticles, including electrochemical etching, electrospinning, sol–gel, chemical vapor deposition, carbothermal reduction, plasma-based techniques, and laser ablation in liquid. Laser ablation in liquid (LAL) is one of the significant methods used to synthesise nanomaterials since it exhibits many advantages over other methods. LAL is simple, eco-friendly, and cost-effective, does not need vacuum and catalysts, exhibits narrow particle size distribution, uniform size and morphology, and constant stoichiometry, *etc.*^8^ The dimensions and morphology of the synthesized nanomaterials strongly depend on many parameters, such as the laser fluence, wavelength, pulse duration, repetition rate, and liquid type. Using the LAL route, nanomaterials can experience high pressures and temperatures, high heating and cooling rates, and short reaction times. Therefore, it is possible to fabricate nanostructures that would be difficult to prepare under normal conditions.^9^ Numerous studies have been reported on the preparation of SiC nanoparticles by the LAL technique. Heidarinassab *et al.*^10^ reported the preparation of 3C silicon carbide (SiC) nanoparticles (NPs) using nanosecond pulsed laser ablation in liquid for electronic and biomedical applications under different conditions. Guo *et al.*^3^ synthesized spherical 4H–SiC with a diameter of 2.48 nm by means of femtosecond laser ablation in deionized water. The authors studied the optical nonlinearity of synthesized SiC nanoparticles. Han *et al.*^11^ adopted an extended non-equilibrium two-temperature model (nTTM) combined with MD simulations to characterize the interactions between the femtosecond laser and 4H–SiC at femtosecond–picosecond scales. SiC nanoparticles have been efficiently used for UV photodetectors, as they can be integrated with crystalline and porous silicon to form high-performance photodetectors.

Arora *et al.*^12^ fabricated a Cu/SiC/Si photodetector, using an RF magnetron, with responsivity of 0.46 A W^−1^ at 440 nm and 1.26 A W^−1^ at 750 nm. Li *et al.*^13^ reported the fabrication of high-performance 4H–SiC NWA MSM UV PDs used to detect 275 and 365 nm UV light, which exhibited a considerable light–dark ratio of 44.3 and extremely high responsivity of 111.9 mA W^−1^ and a detectivity as high as 7.4 × 10^7^ Jones. The fabrication of heterojunction photodetectors based on porous silicon has drawn attention due to their unique optical and electrical properties.^14^ They exhibit many advantages, such as low dark current, high responsivity, broadband response, fast response, no need for high-temperature processing, good linearity, and the ability to operate at high voltages.^15^

In this study, we report a novel approach for preparing SiC nanoparticles *via* the green-route laser ablation of a silicon target in ethanol, which enables silicon carbonization and the *in situ* formation of SiC nanoparticles through a simple and cost-effective process. Furthermore, the fabrication and characterization of a high-performance broadband SiC nanoparticles/porous silicon heterojunction photodetector were successfully demonstrated, and the influence of substrate conductivity type on the photoresponse characteristics of the device was systematically investigated.

## 2. Experimental work

### 2.1 Preparation of porous silicon and colloidal SiC nanoparticles

**Fig. 1:**
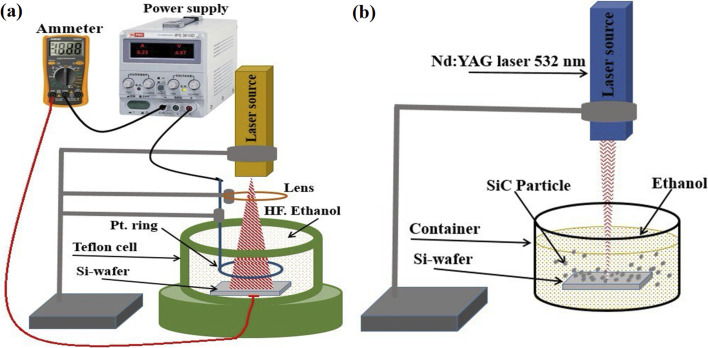
(a) Schematic of the photo-electrochemical etching and (b) pulsed laser ablation in liquid (PLAL) system.

p and n-type porous silicon surfaces were prepared using the laser-assisted electrochemical etching (LAE) method. Single crystals of n-Si and p-Si substrates were obtained with a (100) orientation, an area of 2 cm^2^ and an electrical resistivity of 10 Ω.cm. To prepare the porous silicon, an etching cell consisting of two identical Teflon cylinders was used; the upper cylinder contains a cylindrical cavity containing the etching solution, while the lower cylinder contains a solid column. A silicon wafer was placed on an aluminum foil, and this mixture was deposited on the lower cylinder. The cavity between the two cylinders was filled with the etching electrolyte solution. The positive electrode of the cell was silicon, while the negative electrode was platinum. The cell was filled with a (1 : 1) mixture of HF acid and ethanol. The photoelectrochemical etching process was performed. The etching current density and etching time were selected to be 10 mA cm^−2^ and 5 min, respectively. Fig. 1a shows the schematic diagram of the experimental setup of the photo-electrochemical etching system. After the etching, the substrates were cleaned and stored in a nitrogen atmosphere. A 650 nm, 5 mW laser diode was used to illuminate the silicon substrate during electrochemical etching to prepare (n,p)-type porous silicon. Fig. 1b shows the pulsed laser ablation in liquid (PLAL) system used to synthesize colloidal nanoparticles of SiC. The SiC nanoparticle colloid was prepared by placing a crystalline silicon wafer at the bottom of a glass vessel containing 5 mL of high-purity (99%) ethanol solution, and irradiated with a pulsed laser with a wavelength of 532 nm, an energy of 50 mJ corresponding to a laser fluence of 2.2 J cm^−2^, repetition frequency of 10 Hz, pulse duration of 7 ns, and 500 laser pulses. The laser pulses were focused on the silicon substrate using a converging lens with a focal length of 8 cm.

### 2.2. Characterization of SiC NPs

The structural properties of SIC colloidal nanoparticles were examined using X-ray diffraction (Cu Kα radiation, *λ* = 0.154060 nm), XRD-6000 AS(3K, NOPC) generated at 30 kV and 30 mA in the diffraction range between 10° and 80°. The surface morphology of the nanoparticles was investigated using a field-emission scanning electron microscope (FE-SEM Inspect F50) operating at 30 kV, equipped with energy-dispersive X-ray (EDX). The optical absorption of the colloid was measured using a Shimadzu 1800 double-beam UV-vis spectrophotometer. A Fourier transform infrared (FTIR) spectrophotometer (PerkinElmer, USA) with a spectral resolution of approximately 1 cm^−1^ was employed to study the chemical assignments of SiC nanoparticles. The electrical conductivity and mobility of nanostructured SiC were measured by means of the Hall effect, a light source with variable intensities, a 50% beam splitter, a Jobin Yvon monochromator, and a Sanwa silicon power meter. The effect of bias voltage on the figures of merit of the photodetectors was investigated. Raman spectroscopy measurements were carried out using a Bruker Senterra Raman microscope (Germany) equipped with excitation wavelengths of 532 nm and 785 nm.

### 2.3. Fabrication and characterization of SiC NPs/(p,n) PSi photodetector

**Fig. 2:**
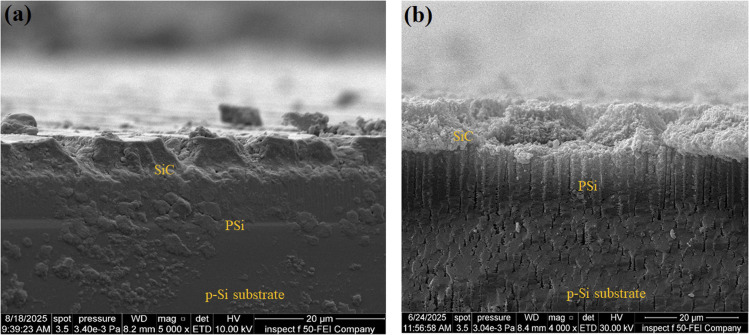
(a) SEM cross-sectional image of SiC/n-Psi and (b) SEM cross-sectional image of the SiC/p-PSi heterojunctions.

To fabricate SiC NPs/(n,p) PSi photodetectors, SiC colloidal NPs were embedded in p-type and n-type porous silicon substrates using a drop-casting method under the same conditions. These conditions include nanoparticle concentration, volume of solution used for drop-casting, and the duration for which the solution was left to dry on the substrate. Fig. 2 shows the SEM cross-sectional images of SiC-(n,p) PSi, which reveal the SiC nanoparticles distributed over the porous silicon layer and some of the nanoparticles embedded inside the pores. Ohmic contacts on the SiC NPs-embedded porous silicon and the backside of the silicon substrate were made by evaporating thin films of indium and aluminum, respectively, as electrodes through an interdigitated metal mask, using a thermal evaporation system. Silver paste was used for wiring the photodetector to the power supply and electrometer.^16^ The optoelectronic and photoresponse properties of the SiC/PSi heterojunction, including dark and illuminated current–voltage characteristics, linearity, responsivity, and detectivity, were measured using a photodetector evaluation system (PES). The main parts of the PES are a Farnell DC power supply, a digital electrometer, a halogen lamp, a monochromator, a beam splitter, and a silicon power meter.

## 3. Results and discussion

**Fig. 3:**
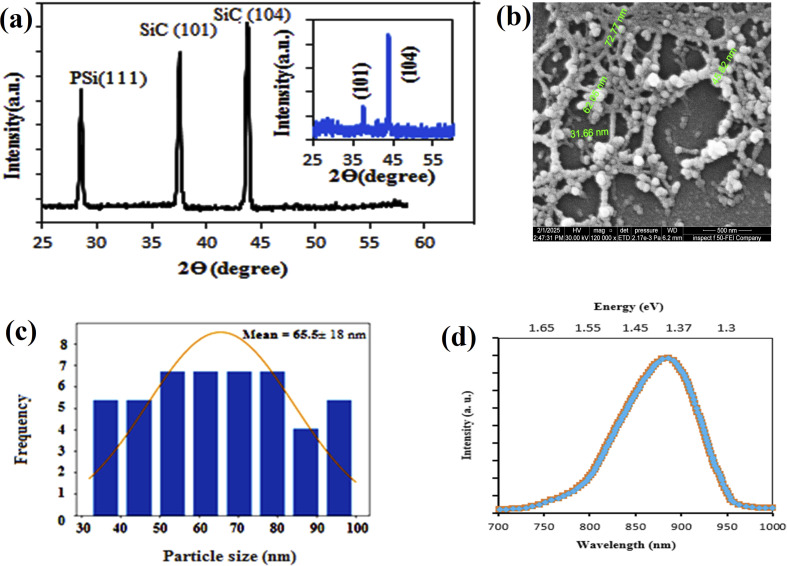
(a) X-ray diffraction pattern of the SiC NPs deposited on Psi, with the inset showing the X-ray diffraction pattern of the SiC deposited on a glass substrate. (b) SEM image of the SiC NPs deposited on a glass substrate. (c) Particle size distribution and (d) photoluminescence spectrum of Psi.

Fig. 3a shows the XRD pattern of SiC NPs deposited on a porous silicon substrate. Three peaks located at 2*θ* = 28.52°, 37.56°, and 43.88°, corresponding to (111), (101), and (104) planes, respectively, were observed. The first peak is related to the porous crystalline silicon substrate, according to JCPDS # 27-1402, and the other two peaks belong to crystalline hexagonal α-SiC, which matched with JCPDS # 29-1131. No XRD peaks related to graphite carbon were detected. The inset of Fig. 1 is the XRD pattern of SiC NPs deposited on a glass substrate. Two XRD peaks were observed at 2*θ* = 37.6° and 43.8°, corresponding to (101) and (10) planes, respectively. Compared to the XRD pattern of SiC NPs deposited on PSi, the intensity at 2*θ* = 43.8° was much higher than that at 37.6°, suggesting a preferred orientation of SiC NPs along the (104) plane. The average crystallite size (*D*) of the SiC NPs deposited on PSi was calculated using the Scherrer formula as follows:1
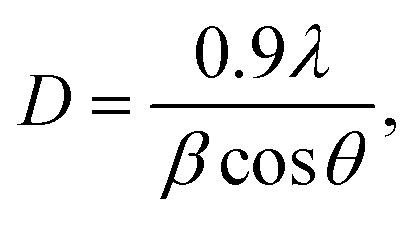
where *λ* is the wavelength of the Cu Kα source (0.15406 nm), *β* is the full width at half maximum, and *θ* is the diffraction angle. The lattice strain (*δ*) and dislocation density (*ε*) of the synthesized SiC NPs were determined from the following equations:2
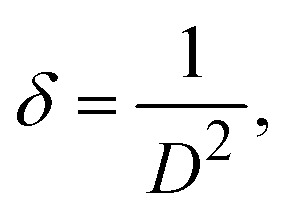
3
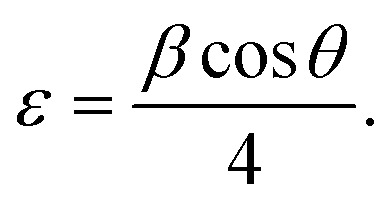


**Fig. 4:**
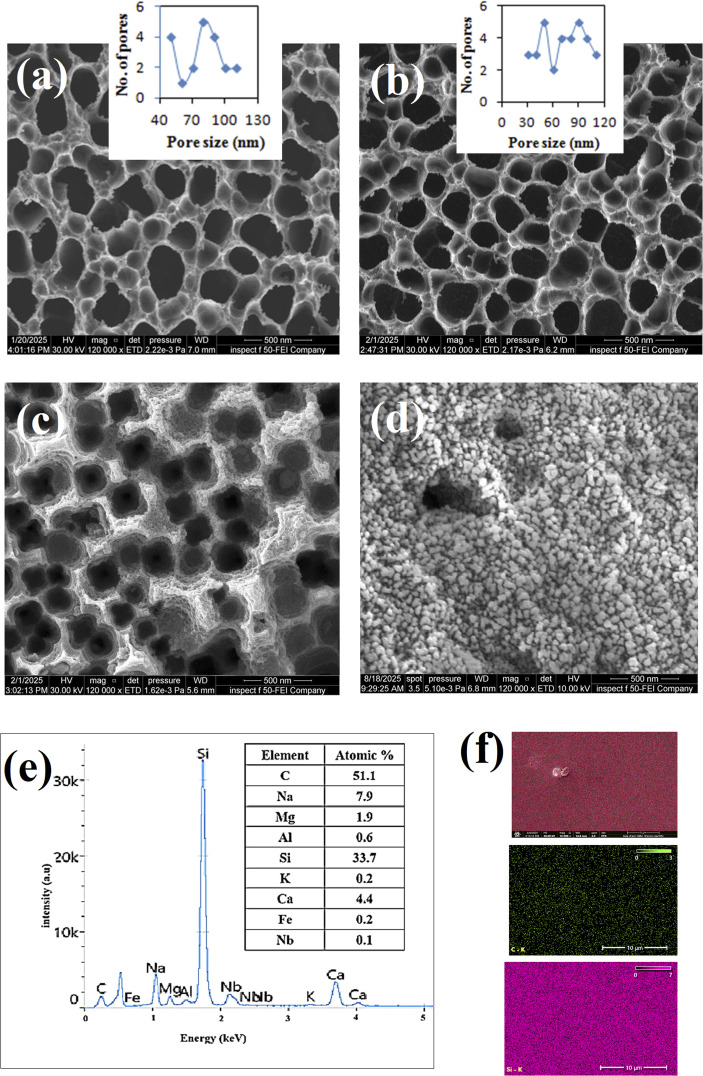
(a) SEM image of n-PSi. Inset is the pore size distribution (b) SEM image of p-PSi. Inset is the pore size distribution (c) SEM image of SiC NPs-embedded n-P-Si, and (d) SEM image of SiC NPs-embedded p-Psi. (e) EDX spectrum of SiC and (f) EDX elemental mapping images of SiC.

The values of crystallite size, strain, and dislocation density are listed in Table 1. The average crystallite size was estimated to be 38 ± 4 nm. Fig. 3b illustrates the SEM image of SiC NPs prepared on a glass substrate. The surface morphology of the nanoparticles exhibits the formation of a granular structure consisting of semi-spherical SiC nanoparticles distributed over the entire surface of the substrate. As shown, some agglomerated nanoparticles were observed because of a high surface energy and van der Waals attractive forces. The observed particles have sizes in the range of 30–70 nm with a mean value of 66 ± 18.7 nm, suggesting that the synthesized SiC NPs have a relatively narrow size distribution (see Fig. 3c). The crystallite size determined by XRD is smaller than the particle size (∼66 nm) observed in SEM. This difference can be attributed to the fact that XRD measures the size of crystallites, while SEM reflects the overall particle size. The larger particle size suggests that each particle may consist of multiple crystallites, indicating a polycrystalline nature. In addition, some degrees of agglomeration during synthesis or sample preparation may also contribute to the formation of large particle sizes observed in SEM. Fig. 4d shows the photoluminescence PL spectrum of porous silicon, which indicates that a broad PL peak was observed at a wavelength of 885 nm corresponding to 1.4 eV.

**Table 1:** XRD analysis of the SiC NPs deposited on Psi

2*θ* (°)	FWHM (°)	*d*-Observed (Å)	Crystallite size *D* (nm)	Microstrain (*ε*)	Dislocation density (nm^−2^)
37.6	0.285	2.390	35	3.66 × 10^−3^	1.16 × 10^−5^
43.8	0.349	2.065	41	3.79 × 10^−3^	1.67 × 10^−5^

The SEM images of n-PSi and p-PSi are shown in Fig. 4a and b. The images confirm the formation of a porous structure with semicircular pores. The porosity percentage of (p,n) PSi calculated from SEM images with the aid of the ImageJ software was found to be 55.1% for n-PSi and 55.8% for p-PSi. The pore size distributions of n-PSi and p-PSi are shown in the inset of Fig. 3a and b. As shown, the porous layers consist of pores of different sizes. The mean pore size distributions of n-PSi and p-PSi were 85 nm and 73 nm, respectively. Fig. 3c and d show the SEM images of SiC NPs deposited on n-PSi and p-PSi, respectively. As shown in Fig. 4c, the SiC nanoparticles were embedded inside the pores, demonstrating strong infiltration into the porous matrix. On the contrary, for SiC nanoparticles deposited on n-PSi, as shown in Fig. 4d, the nanoparticles were found to be attached to the pore walls and surface, with limited penetration into the pores. We can attribute these findings to the fact that for the p-PSi sample, the embedding of SiC nanoparticles inside the pores arises from the difference in surface charge between the SiC (negative charge and the positive charge of the p-type), which prompts nanoparticle infiltration into the pores. For the n-PSi sample, the electrostatic repulsion formed between SiC NPs and n-PSi limits penetration and favors the surface deposition of the SiC NPs. Other factors may also contribute to this result, including band bending, wettability, and the morphology of the pores. Fig. 3e depicts the EDX spectrum of SiC-deposited porous silicon. The peaks of both Si and C elements are observed, which represent the main elements of the SiC NPs, and the other peaks belong to the glass substrate elements. As shown in the inset of Fig. 4e, the atomic percentage ratio of the product [Si]/[C] = 0.66, indicating the formation of non-stoichiometric SiC. This result can be attributed to a very fast laser quenching process with insufficient time for the ordering of Si–C, as well as the decomposition of ethanol during laser irradiation, which could cause the formation of the carbon-rich product SiC_*x*_. The EDX elemental mapping of SiC NPs shown in Fig. 4f confirms the presence of C and Si elements, and the relative concentration of C was found to be higher than that of Si.

**Fig. 5:**
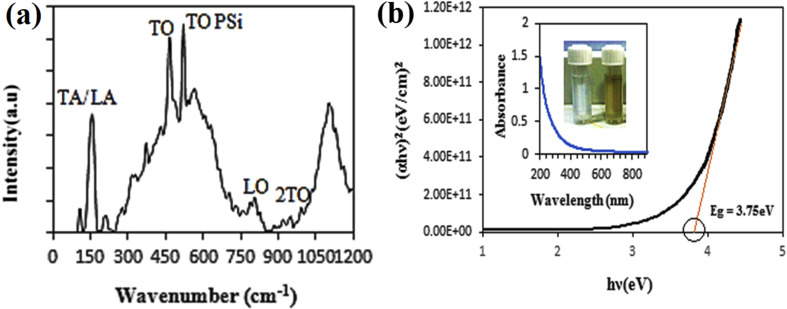
(a) Raman shift spectrum and (b) (*αhν*)^2^*versus* (*hν*) plot of the SiC colloid. The insets show the optical absorbance plot and a photograph of the SiC colloids.

Fig. 5a shows the Raman spectrum of SiC NPs deposited on porous silicon. All these Raman peaks belong to hexagonal α-SiC. As shown in Table 2, the peak observed at 157 cm^−1^ is indexed to TA/LA acoustic modes and the peak located at 463 cm^−1^ is attributed to the TO mode of Si–C. The peak observed at 520 cm^−1^ is related to the TO of porous silicon. A peak at 783 cm^−1^ was observed, which can be indexed to the LO mode of Si–C. A strong broad peak was detected in the range 950–1150 cm^−1^, which can be indexed to second-order SiC modes.^17–21^

**Table 2:** Raman peaks of the SiC NPs

Wavenumber (cm^−1^)	Assignment
157	TA/LA acoustic modes
270–300	Second-order acoustic (LA + TA)
463	TO (transverse optical) mode of Si–C
520	TO of porous silicon
783	LO (longitudinal optical) mode of Si–C
950–1000	Second-order Raman modes (2TO or TO + LO)
1100–1150	Second-order SiC modes/possible Si–O–Si

The optical energy gap of the SiC colloidal NPs was determined using a Tauc plot, according to the following formula:

4 (*αhν*)^2^ = *A*(*hν* − *E*_g_),

where *α* is the absorption coefficient of SiC and *A* is a constant that depends on the material type. Fig. 5b illustrates the variation of (*αhν*)2 with photon energy (*hν*). The energy gap can be obtained by extrapolating the fitted linear part of the second region of the curve to the (*αhν*)^2^ = 0 point. As shown in Fig. 4b, the optical energy gap was found to be *E*_g_ = 3.75 eV, which is larger than that of bulk α-SiC (3.0–3.3) eV due to the quantum size effect (QSE, nanosized particles), phonon and carrier confinement, size-dependent optical modeling, and increased surface-to-volume ratio. This, in turn, leads to quantization of the energy levels of SiC and a widening of its energy gap. The insets of Fig. 5b are the optical absorbance spectrum and a photograph of the SiC colloid. The absorbance decreased after *λ* = 200 nm due to the absorption edge of SiC, as well as the semiconducting nature of SiC. The color of the ethanol changed from transparent to light yellowish after laser ablation, indicating the formation of SiC NPs. We think the carbon-rich deviation from the ideal stoichiometry of SiC is expected to introduce defect states, such as vacancies, within the material. These defects, in turn, can modify the optical properties of SiC NPs by modifying the band structure and introducing localized states in the bandgap. Consequently, they may affect charge carrier transport and recombination dynamics, in addition to altering the optical absorption behavior of the material.

**Fig. 6:**
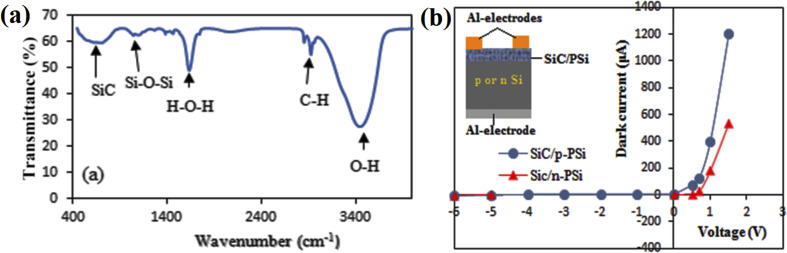
(a) FT-IR spectrum of the SiC colloid. (b) Dark *I*–*V* characteristics of the n-SiC/p-PSi and n-SiC/n-PSi heterojunctions. The inset shows the schematic of the cross-section of the heterojunction with the electrical configuration.

The FT-IR spectrum of SiC NPs illustrated in Fig. 6a shows characteristic peaks of SiC, along with other bonds resulting from the medium and liquid. The absorption peak observed at 720 cm^−1^ can be attributed to the vibration of the Si–C bond, confirming the formation of silicon carbide. FT-IR peaks observed at 979 cm^−1^ and 1100 cm^−1^ were indexed to stretching Si–O–Si and Si–O bonds, respectively, arising from the trapped oxygen within the porous matrix and may be due to the surface oxidation of the porous silicon. An absorption band at 1662 cm^−1^ is related to the H–O–H cm^−1^ bending vibration, resulting from the absorption of moisture from the atmosphere and/or residues of ethanol after evaporation. A broad peak detected in the range of 3300–3500 cm^−1^ is attributed to the stretching O–H vibration arising from the presence of hydroxyl groups or water molecules absorbed on the surface.

Hall effect measurements revealed that the SiC NPs have a negative Hall coefficient, indicating that SiC is n-type. The electrical resistivity and electron mobility of nanostructured SiC were 2.8 × 10^4^ Ω cm and 72 cm^2^ V^−1^ s^−1^, respectively. The mobility is lower than that of bulk SiC due to the high density of grain boundaries, which restricts the transport of the charge carriers. The dark current–voltage characteristics of SiC NPs/n-PSi and SiC NPs/p-PSi heterojunctions are shown in Fig. 6b, in the voltage range of −5 V to + 1.5 V. They exhibited rectifying behavior; the forward current for both heterojunctions increased exponentially with voltage, and the reverse currents were bias-voltage independent, indicating that they follow the diode equation. The forward current of the n-SiC NPs/p-PSi heterojunction is larger than that of n-SiC NPs/n-PSi and, in turn, exhibits better rectifying properties. This is because in the case of n-Si/n-PSi (n–n interface), the conduction band offset restricts electron transport, and the absence of hole injection produces a higher effective potential barrier that reduces the forward current flow.

**Fig. 7:**
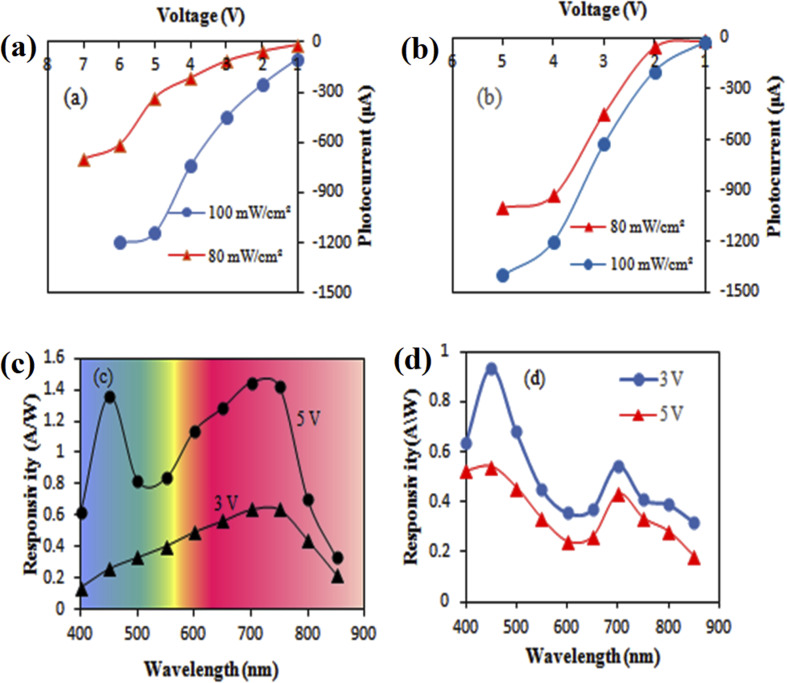
(a) Photo *I–V* characteristics of the n-SiC/n-PSi photodetector and (b) photo *I–V* characteristics of the n-SiC/p-PSi photodetector. (c) Responsivity of the n-SiC/p-PSi photodetector at 3 and 5 V and (d) responsivity of the n-SiC/n-PSi photodetector at 3 and 5 V.

The ideality factor of the SiC/n-PSi and SiC/p-PSi heterojunctions was determined from the diode equation after finding the saturation current from semilogarithmic forward *I*–*V* characteristics, and found to be 4 and 2.4, respectively. The high value of the ideality factor (*n* = 2.4–4) implies non-ideal charge transport in the SiC/P–Si device. This deviation from unity suggests that current conduction in the device is not governed only by thermionic emission but is influenced by additional mechanisms such as recombination in the depletion region, barrier height inhomogeneities at the SiC–PSi interface, and trap-assisted transport through interface defect states. The transport behavior can be explained as a combination of thermionic emission and recombination-dominated processes, confirming the presence of interface-related defects in the device. The photo *I*–*V* characteristics of SiC/n-PSi and SiC/p-PSi heterojunction photodetectors illuminated by white light intensities are shown in Fig. 7a and b. For the SiC/n-PSi photodetector, the photocurrent was measured in reverse bias, where the n-PSi was connected to the positive electrode and SiC to the negative electrode, while for SiC/-p-PSi, the p-PSi was connected to the negative electrode and SiC to the positive electrode.^22,23^ When the photodetector is illuminated, the absorbed photons produce an e–h pair, increasing the current, and the electric field prevents the recombination of these photogenerated carriers. Increasing the light intensity leads to an increase in the photocurrent without any observed significant saturation due to increases in the number of absorbed photons. The photocurrent of the n-SiC/n-PSi (isotype) photodetector is lower than that of the n-SiC/p-PSi (anisotype) photodetector because the latter has a wider depletion layer than the former.^24,25^ The responsivity of the SiC/n-PSi and SiC/p-PSi at two bias voltages photodetectors are shown in Fig. 6c and d. The responsivity *R*_*λ*_ of the photodetector can be calculated from the following equation:5
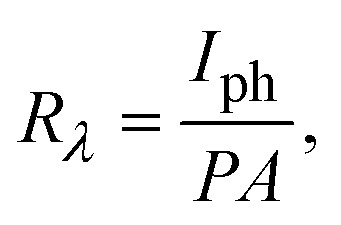
where *I*_ph_ is the photocurrent, *P* is the intensity of the light at a certain wavelength, and *A* is the photosensitive area of the photodetector. The responsivity of anisotype SiC/n-PSi at a bias voltage of −5 V, as shown in Fig. 7c, exhibits two response peaks at 450 nm and 750 nm, with responsivities of 1.36 A W^−1^ and 1.44 A W^−1^, respectively. We attributed the origin of these two peaks to the photons absorbed near the absorption edge of SiC and porous silicon, respectively. The short wavelengths were absorbed in SiC and the longer wavelengths were absorbed in porous silicon. Despite the SiC NPs exhibiting a large optical energy gap of approximately 3.75 eV, corresponding to absorption in the UV region, the photon energy at 450 nm (∼2.76 eV) is not sufficient to induce direct band-to-band excitation in SiC. Consequently, the observed visible-light photoresponse of the SiC/PS heterostructure can be attributed mainly to photocarrier generation within the depletion layer of the porous silicon layer. Basically, the role of the SiC nanoparticles is associated with interfacial engineering effects, including the prevention of carrier recombination, surface passivation of porous silicon defects, and enhancement of charge separation and carrier transport across the SiC NPs/PSi interface. Additionally, structural defects and interface states at the SiC/PSi heterojunction may contribute to sub-bandgap carrier transfer processes under visible illumination.

The photodetector biased to −3 V yielded a lower responsivity because the depletion layer width increases as bias voltage increases, which explains why only one peak of response was observed at a bias voltage of −3 V.

In contrast, the isotype SiC/n-PSi photodetector revealed a lower responsivity of 0.9 A W^−1^ at 450 nm and 0.53 A W^−1^ at 700 nm, compared to that of an anisotype photodetector due to the fact that its depletion layer thickness is smaller than that of SiC/p-PSi, indicating a higher probability for recombination of the photogenerated carriers absorbed outside of the depletion region.^26^

**Fig. 8:**
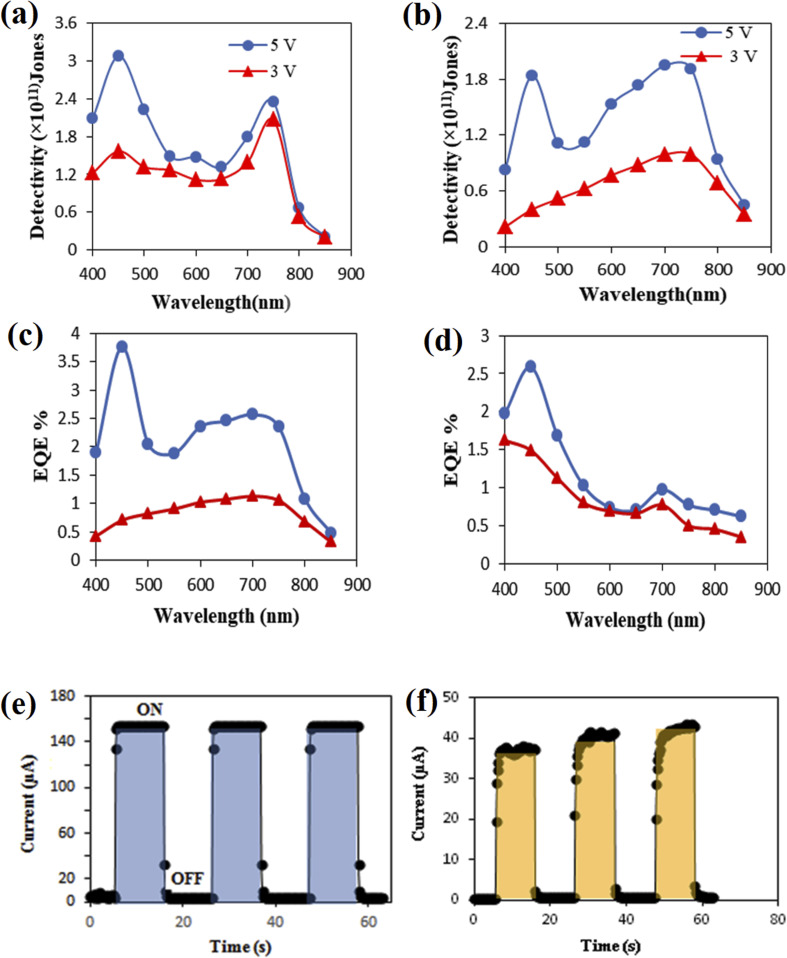
(a) D* of the n-SiC/p-PSi photodetector and (b) D* of the n-SiC/n-PSi photodetector. (c) EQE of the n-SiC/p-PSi photodetector and (d) EQE of the n-SiC/n-PSi photodetector. (e) Photocurrent-time dependence of the n-SiC/p-PSi photodetector. (f) *I–t* properties of the n-SiC/n-PSi photodetector.

The specific detectivity D* plots of the photodetector of n-SiC/p-PSi and n-SiC/p-nSi photodetectors at bias voltages of −3 and −5 V are depicted in Fig. 8a and b. The detectivity was calculated using the following relationship:6
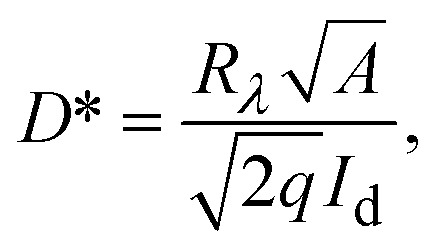
where *A* is the sensitive area of the photodetector, *q* is the electron charge, and *I*_d_ is the dark current. The detectivity of the photodetectors increased as the bias voltage increased from −3 V to −5 V due to the widening of the depletion region, and consequently enhanced the separation of photogenerated carriers that were generated outside of the depletion region.^9,27,28^ This increased the swept efficiency of the charge carriers toward the electrodes and contributed to increasing the photocurrent. The D* of the n-SiC/p-PSi and n-SiC/n-PSi photodetectors was 3.07 × 10^11^ Jones and 1.83 × 10^11^ Jones at 450 nm at −5 V, respectively. We attributed this result to the large thickness of the depletion region and the high built-in electric field of n-SiC/p-PSi compared to the n-SiC/n-PSi photodetector. In the latter photodetector, the two layers are electron-conducting only, and the built-in electric field comes from the difference in their energy gaps.^29–31^Fig. 8c and d illustrates the external quantum efficiency (EQE) of the photodetectors at bias voltages of −3 V and −5 V. As shown, the maximum value of the EQE was (3.75 × 10^2^)% and (2.58 × 10^2^)% at 450 nm for SiC/p-PSi and SiC/n-PSi photodetectors at −5 V, respectively. The value of EQE exceeds unity, which can be ascribed to the formation of internal gain. The high density of surface and trap states in the porous silicon structure effectively prolonged the carrier lifetime with respect to the transit time (*τ* > *t*_r_), enabling one photogenerated charge carrier to contribute to multiple charge collections. Fig. 8e and f shows the time-dependent photocurrent response plot of the n-SiC/n-PSi and n-SiC/p-PSi photodetectors under periodic ON/OFF switching of light pulses.

As is clearly shown, the photocurrents of both photodetectors show repeatable and fast switching behavior, confirming that the fabricated photodetectors have good photoresponse and device reliability.

**Fig. 9:**
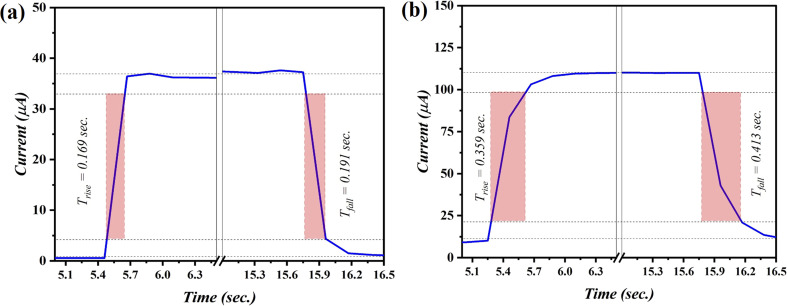
Rise and fall times of the (a) SiC/p-PSi and (b) SiC/n-PSi photodetectors.

Upon illumination (ON-state), the photocurrent increases (generation of the carriers larger than recombination, G > R) and then reaches steady state (G = R). When the light is turned off (OF-state), the current drops to dark current (R > G). The sharp contrast between the ON-state and OFF-state indicates the efficient separation of photogenerated charge carriers. The rise time/fall time ratios (*τ*_r_/*τ*_f_) of the p-SiC/p-PSi and n-SiC/n-PSi photodetectors were determined and found to be 248 ms/306 ms and 168.8 ms/190.9 ms, respectively, as shown in Fig. 9, and the corresponding ON/OFF current ratio was 137 and 30. Based on the results obtained, n-SiC/n-PSi is faster than the n-SiC/p-PSi photodetector because in the former, there is only one type of charge carrier (electrons), which has higher mobility than holes, leading to a shorter path length and, consequently, a faster response.

**Fig. 10:**
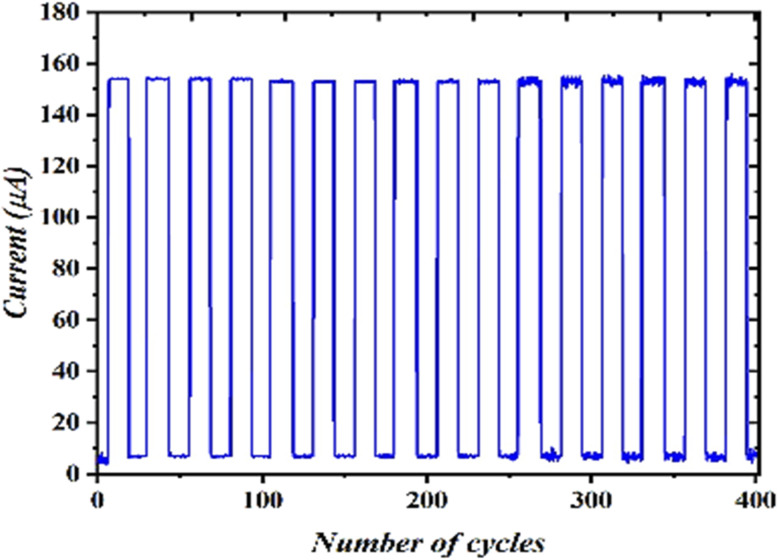
Stability and repeatability plot of the n-SiC/p-PSi under periodic light ON/OFF switching.

Fig. 10 illustrates the long-term cycling stability of the photodetector evaluated over approximately 400 ON–OFF cycles. The device exhibits highly stable and reproducible photoresponses, with negligible variation in both the photocurrent (ON-state) and dark current (OFF-state) throughout the measurement. No remarkable degradation in the ON-state current is observed, confirming the excellent operational stability and reversibility of the fabricated photodetector.

**Fig. 11:**
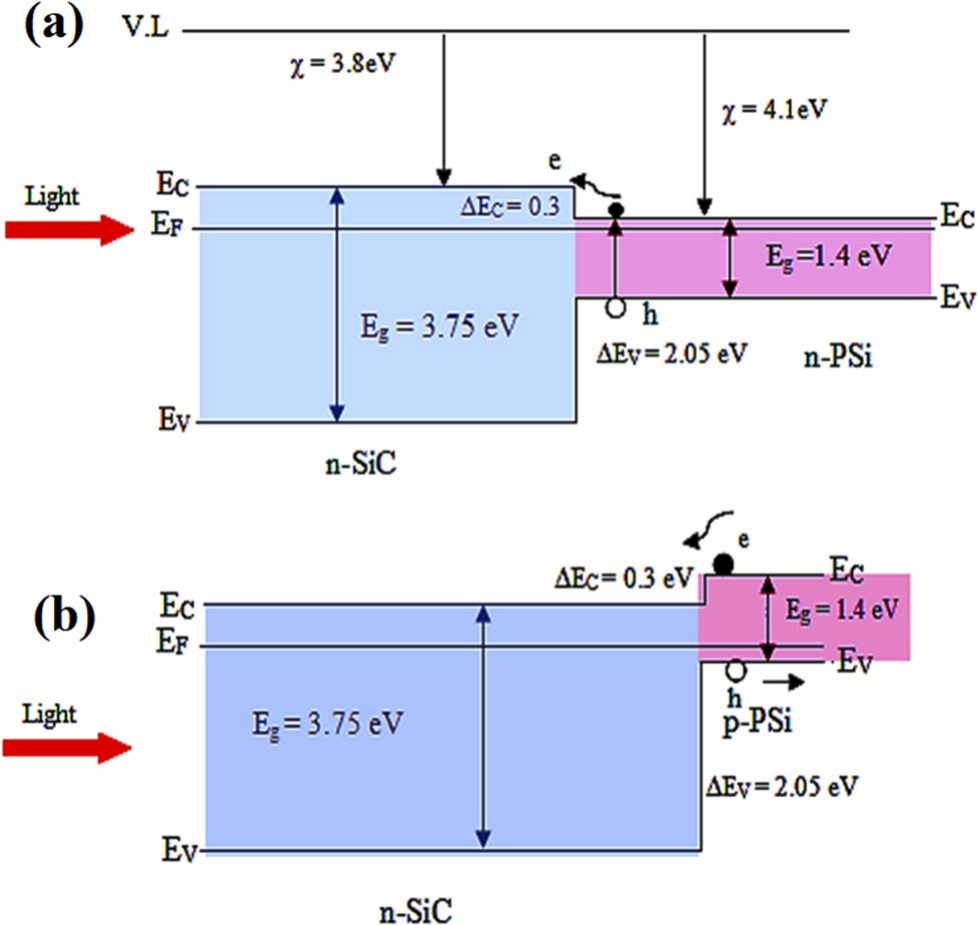
Energy band diagrams of the (a) n-SiC/n-PSi photodetector and (b) n-SiC/p-PSi photodetector under illumination.

The energy band diagrams of the SiC NPs/PSi heterojunctions under illumination are shown in Fig. 11. The band offsets of n-SiC/PSi were calculated to be Δ*E*_c_ = *χ*(PSi) − *χ*(SiC) = 4.05 − 3.75 = 0.3 eV and Δ*E*_V_ = *E*_g_(SiC) − *E*_g_(PSi) − Δ*E*_c_ = 3.75 − 1.4 − 0.3 = 2.05 eV. The position of the Fermi energy level in SiC was *E*_C_ − *E*_F_ = 0.4 eV, and that for PSi was *E*_F_ − *E*_V_ = 0.2 eV. In the n-SiC/n-PSi heterojunction, the mechanism of photodetection is governed by the band offsets at the (n)SiC–(n)PSi interface rather than by a strong built-in electric field. When the photodetector is illuminated with photons with energy *hν* < *E*_g_, the electron–hole pairs are produced due to the photon's absorption in the porous Si layer due to its smaller energy gap. The photogenerated electrons are transferred efficiently across the interface into the n-SiC layer due to the small value of the conduction band offset (Δ*E*_C_ = 0.3 eV), resulting in an increase in photocurrent. Nevertheless, the presence of a weak internal electric field leads to limited e–h separation, and photogenerated holes recombine at defect states within the porous structure or at the interface. Consequently, although the n–n heterojunction can display a fast photoresponse and reasonable photocurrent, it exhibits higher noise current and moderate specific detectivity. In contrast to the (n–n) SiC/PSi heterojunction, the mechanism of photodetection of the (p–n) SiC/PSi heterojunction is dominated by the charge carrier separation by the internal electric field of the depletion region. Upon illumination, electron–hole pairs are generated in the depletion region of the porous p-Si layer, and due to the presence of a strong internal field, they are separated so that the electrons are driven toward the SiC, whereas holes are transported toward the p-PSi side. The small value of Δ*E*_C_ may facilitate electron extraction, whereas the large value of Δ*E*_V_ (Δ*E*_V_ = 2.05 eV) efficiently prevents hole back-diffusion and, in turn, suppresses interfacial e–h recombination. The separation of the photogenerated charge carriers by a strong electric field leads to obvious rectifying behavior,^32^ decreasing dark current, improved responsivity, and higher specific detectivity compared to the n–n heterojunction. These findings are consistent with the results of responsivity, quantum efficiency, response time, and specific detectivity of the (n–n) SiC/PSi and (p–n) SiC/PSi photodetectors.

Table 3 lists the figures of merit of the fabricated SiC/n-PSi and SiC/p-PSi photodetectors compared with those of reported heterojunction-based silicon photodetectors. This table confirms that the fabricated SiC/p-PSi photodetector exhibits higher responsivity and detectivity in the visible and near-IR regions than the reported SiC/PSi photodetectors. This finding can be attributed to the optimized structural, optical, and interfacial properties of the SiC/PSi heterostructure.

**Table 3:** Comparison of the photodetector parameters with those of some reported silicon photodetectors

Photodetector type	Preparation method	Responsivity (A W^−1^)	Detectivity (D*) (Jones)	EQE (%)	References
4H–SiC/p-Si heterojunction	Laser ablation in ethanol (Nd:YAG, 1064 nm)	2.5	2.5 × 10^14^	397	9
SiC/porous Si (PLD)	Pulsed laser deposition (Nd:YAG, 1064 nm)	0.0096 at 365 nm	2.45 × 10^10^	340	33
p-3C–SiC/p-Si/n-Si (double junction)	Epitaxial growth of 3C–SiC on Si + Al sputtering	8.83 × 10^−2^	2.25 × 10^10^	17.3	34
n-3C–SiC/p-Si heterojunction	Epitaxial growth of 3C–SiC on Si + photolithograph	—	6.9 × 10^12^	—	35
GaN/porous Si heterojunction	Pulsed laser deposition (Nd:YAG, 355 nm)	29.03 at 370 nm	8.6 × 10^12^	97.2	36
n-SiC NPs/p-PSi	Laser ablation of Si in ethanol (Nd:YAG, 532 nm)	1.44 at 450 nm	3.3 × 10^11^ at 450 nm	380 at 450 nm	This work
SiC NPs/n-PSi		0.95 at 450 nm	2.3 × 10^11^ at 750 nm (3 V)	257 at 450 nm	

## 4. Conclusion

High-performance broadband SiC NPs/PSi heterojunction photodetectors were successfully fabricated using the pulsed laser ablation of silicon in ethanol, followed by electrochemical etching. The synthesized SiC nanoparticles exhibited a spherical morphology and were embedded inside the pores of porous silicon in the case of p-type PSi, while they were mainly deposited on the pore walls for n-type PSi. The SiC NPs/p-PSi photodetector exhibited high responsivity and specific detectivity, which comes from enhanced carrier separation and a stronger built-in electric field. In contrast, the SiC NPs/n-PSi device was found to be faster than the SiC NPs/p-PSi photodetector. The SiC NPs/p-PSi photodetector achieved a responsivity as high as 1.4 A W^−1^ at 450 nm, a detectivity of 3.07 × 10^11^ Jones, and an external quantum efficiency of (3.75 × 10^2^)%. The illuminated energy band diagram confirms that the photoresponse of the SiC NPs/p-PSi photodetector is more efficient than that of the SiC NPs/n-PSi device. Based on the results obtained, the proposed SiC NPs/PSi heterojunction offers a promising route for the fabrication of low-cost, visible-enhanced photodetectors *via* an eco-friendly synthesis approach.

## Conflicts of interest

The authors declare that they have no known competing financial interests or personal relationships that could have appeared to influence the work reported in this paper.

## Data Availability

The datasets generated during and/or analyzed during the current study are available from the corresponding author (R. A. Ismail) upon reasonable request.
